# The mediating roles of demand and satisfaction in formation process of physical exercise habits among college students

**DOI:** 10.1038/s41598-022-05602-3

**Published:** 2022-01-28

**Authors:** Kun Wang, Jiali Qian, Jiayi Yang, Tianyi Ge, Zhizhong Li

**Affiliations:** Department of Physical Education, Shanghai Jiao Tong University, 204 Guangming Hall, NO. 800 Dongchuan Street, Minhang District, Shanghai, 200240 China

**Keywords:** Human behaviour, Lifestyle modification

## Abstract

Considering the situation and disadvantages of being physically inactive as well as the nature and advantages of doing physical exercise regularly, there is a need to explore how physical exercise habits are cultivated and formed. The study was to examine the formation process of physical exercise habits. According to the Model of Physical Exercise and Habit, It was speculated that satisfaction, demand or chain from satisfaction to demand could mediate the relationship between physical exercise behavior and physical exercise habit. Cross-sectional design with 3202 college or university students from China was employed. Data about physical exercise habits, physical exercise behaviors as well as related questions was measured by the Self-Report Exercise Habits Index and direct questions. Structural Equation Modeling (SEM) was constructed to evaluate the mediating effects of demand and/or satisfaction by Asymptotically Distribution-Free and BOOTSTRAP. The inferential statistics was to estimate path coefficient and mediation effect. Findings suggested physical exercise behaviors could develop into physical exercise habits through a direct path, single mediators of demand or satisfaction, or a chain mediator from demand to satisfaction.

## Introduction

It has been documented that doing physical exercise regularly could improve individuals' cognition flexibility^1^, muscle strength and endurance^2^ and reduce the incidence of cardiovascular disease, type 2 diabetes and depression^3^, thereby helping to keep healthy and prompting quality of life^4^. However, news on the World Health Organization (WHO) website showed that only 22% boys and 15% girls in the world do exercise sufficiently^5^ and the matter was approximately 5 million deaths worldwide every year just because of being physically inactive^6^. To promote healthy development of our body and mind, how to enhance physical exercise behaviors and form physical exercise habits seem to be major issues. Developing and maintaining a habit is not effortless but needs to be cultivated and developed rationally, especially for physical exercise behaviors due to the fact that doing physical exercise is often perceived as a sweatful and tedious activity and makes individuals shy away from it.

Similar to the definition of “habit”^7^, physical exercise habits refer to positive, stable, and automated behavioral and thinking patterns through repeated exercise practices in a specific situation^8^. The characteristics of it are mainly about repetition and automatic^9,10^ which has less effort and awareness^11,12^.

Thus far, some scholars believed that motivation was one of the most vital aspects of forming physical exercise habits or physical exercise adherence^13,14^. In line with the Self-Determined Theory, different motivation (i.e. internal motivation, external motivation and amotivation) resulted in positive or negative intentions to physical exercise habits through different regulations^15^. Kilpatrick et al. held the opinion that external motivation, such as weight-losing or stress-managing, and intrinsic motivation, such as enjoyment and challenge, were influential factors in sports participation and they were more likely to be causes in persistence of doing physical exercise^16^. Gardner and Lally also noted that self-determined regulation had an impact on habit strength^17^. Nevertheless, Magaraggia et al. concluded that exercise frequency was not affected by motivation^18^. Since frequency or repetition of a behavior were necessary parts of a habit formation^19^, physical exercise behavior itself was paid more attention to when forming physical exercise habits. Kaushal and Rhodes proposed that doing physical exercise at least four times a week for about six weeks could help form physical exercise habits^20^. Harris and Kessler designed intervention experiments and proposed that the higher frequency and the longer duration of the initial stage in several consecutive weeks had positive causality for the possibility of persisting to do physical exercise^21^. At the same time, flexible incentives^22^, regular feedback^23^ and positive affective states^24^ could help maintain physical exercise adherence. A review on the influencing factors of physical exercise behaviors (habits) believed that physical exercise behaviors (habits) were chosen for various reasons^25^, such as perception of competence^26,27^, fun and enjoyment^28,29^, parents^30^, learning new skills^31^, friends and peers^32^. Studies proposed that the exercising types of adolescents' were related to the views of their parents^33^, and satisfaction from children with their exercising experience depended on support and encouragement of their parents^34,35^. Except the sparse articles on physical exercise habits, more research was about exercise adherence on unhealthy populations, such as populations with persistent musculoskeletal pain^36^ or older adults suffering from chronic knee pain^37^. Even though unhealthy populations were delicate and vulnerable, there was still a need to help normal people form positive physical exercise habits to maintain and promote their physical and mental function.

**Figure 1:**
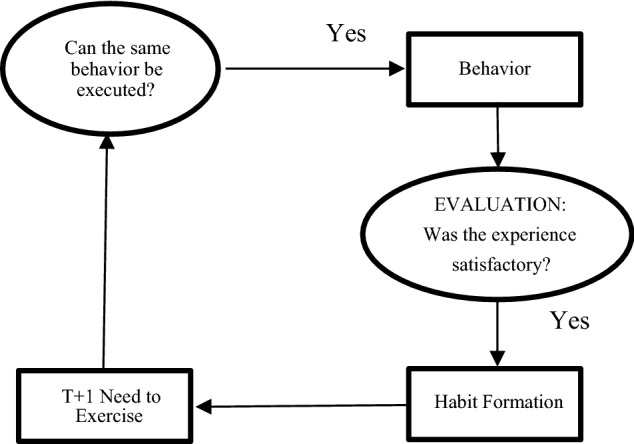
Formation Processes of a (Physical Exercise) Habit.

Generally speaking, some psychological research on behavior and habits relied on the rational choice model. The attitude-behavior model by Fishbein and Ajzen, named Theory of Reasoned Action as well, believes that attitudes which representing the desire for behaviors after trading-off positive and negative consequences of individuals, and subjective norms which representing social pressure experienced before, lead to behavioral intention, and then tend to behavior^38^. Later, Perceived Behavior Control was added as the third concept of behavior prediction in the model and the Theory of Planned Behavior was developed^39^. Noteworthy is the fact that Perceived Behavior Control is close to the Belief of Self-Efficacy in Bandura’s Social Learning Theory, which both account for the perception of difficulty when conducting a behavior^40^. However, the models above ignored the repetitive nature of physical exercise behaviors^10^ and the dynamic nature of physical exercise habits formation that whether to do physical exercise again was mostly influenced by previous exercising experience^41^. Considering the theoretical background above, Aarts put forward the Model of Physical Exercise and Habit Formation ^10^. Through the Theory of Reasoned Action and the Theory of Planned Behavior, behavior has come into being, and then the bold arrows are the formation processes of a habit (Fig. 1)^10^. According to the Model of Physical Exercise and Habit Formation, when the same needs reappear, individuals could recall their experiences of physical exercise. If they were satisfied, individuals would be more willing to repeat the same behaviors^10^. After repeating too many times, cognition shortcuts, operationally defined as reactions to the situation automatically without deliberating, will come out and habits will be formed lastly^10^.

Given the situation and disadvantages of being physically inactive, the nature and advantages of doing physical exercise regularly as well as the gap of previous research, taken together, the purpose of this research was to examine the formation processes of physical exercising habits (Fig. 1). Based on the Model of Physical Exercise and Habit Formation, three hypotheses were given:

### H1

Satisfaction could mediate the relationship between physical exercise behavior and physical exercise habit.

### H2

Demand could mediate the relationship between physical exercise behavior and physical exercise habit.

### H3

Chain from satisfaction to demand could mediate the relationship between physical exercise behavior and physical exercise habit.

## Methods

### Participant

Cross-sectional design with convenient samples was employed in this study. All participants were recruited via online survey randomly. Participants were selected as who: (1) were university or college students; (2) were able to read and understand Mandarin language and the purpose of this survey; and (3) were native Chinese.

Questionnaires about their physical exercise behavior, physical exercise habits and other related questions were distributed to eligible participants. Initially, 3916 questionnaires for this survey were obtained. In the end, 3202 samples from 34 provinces and regions in China with an effective rate of 82% were retained upon rigorous exclusion criteria. The exclusion criteria were which questionnaires: (1) were answered within 50 s; (2) had missing answers on items; and (3) had regularity or consistency in their answer. The demographic of participants was as follows (Table 1).

**Table 1:** Demographic of participants.

		N	%	M	SD
Age	Total	3202		19.45	1.58
	Male	2084	65%	19.44	1.54
	Female	1118	35%	19.49	1.66
Gender	Male	2084	65%		
	Female	1118	35%		
Nationality	Han	2948	92%		
	minority	254	8%		
Region	Shanghai	504	15.7%		
	North China	713	22.3%		
	South China	611	19.1%		
	East China	636	19.9%		
	West China	707	22.1%		
	Macao, Hongkong, Taiwan	31	0.01%		

### Study design

Cross-sectional design was conducted in this study. Data was collected through commercial online survey platforms (QR code from Wenjuanxing and Wechat). Demographic information (i.e. age, gender, nationality, original place) was collected at the beginning of the survey. The surveys about physical exercise behavior, demand of doing physical exercise, satisfaction of exercising experience and physical exercise habit were followed. Details of the questionnaire were reported in measurements.

The questionnaires were distributed to participants in a voluntary and independent approach and the contents were totally anonymous, strictly confidential and solely research-used. Participants were provided informed consent. The study had been approved by the local ethics committee of the Institional Review Board for Human Research Protections at Shanghai Jiao Tong University, China (H20200431I) and accorded with the guidelines of the Declaration of Helsinki.

### Measurements

Physical exercise behaviors, demand and satisfaction.

Physical exercise behaviors, demand and satisfaction were measured by direct questions, including:

- "I have physical exercise behaviors " (referred to as "physical exercise behavior");
- "I will execute physical exercise again for the same demand, like losing weight" (referred to as "demand ");
- "I was satisfied with my physical exercise experiences" (referred to as "satisfaction").

To echo the true meaning of what the model expresses, questions in the model were conducted directly. 1–5 Likert scale was conducted (1 = completely inconsistent; 5 = completely consistent). The higher the score was, the more consistent the reality was with the statement.

Physical exercise habits.

Physical exercise habits were assessed using the Self-Report Exercise Habits Index (SREHI). It was modified from the Self-Report Habits Index (SRHI) with physical exercise scenarios. Initially, SRHI was contextualized with physical exercise scenarios and revised to SREHI. Then, SREHI was translated into Mandarin by three native Mandarin speakers and the Mandarin items were back-translated into English by three English translators to evaluate the consistency between the English version and Mandarin version. Lastly, the final version was double-checked by an expert in the field to ensure the accuracy of SREHI.

Two principal components were extracted and the cumulative contribution rate (Initial Eigenvalues) was 78.77% (KMO = 0.95, *p* < 0.001). Two dimensions, named as automaticity (4 items) and repetition (3 items), accorded with the two characteristics of habits by Aarts et al., automated response to a specific situation and tendency to repeat the same behavior^10^. 1–5 Likert scale (1 = completely inconsistent; 5 = completely consistent) was conducted to each item and total scores of automaticity, repetition and physical exercise habits were calculated. The higher the score was, the more stable automaticity, repetition and physical exercise habits were.

**Figure 2:**
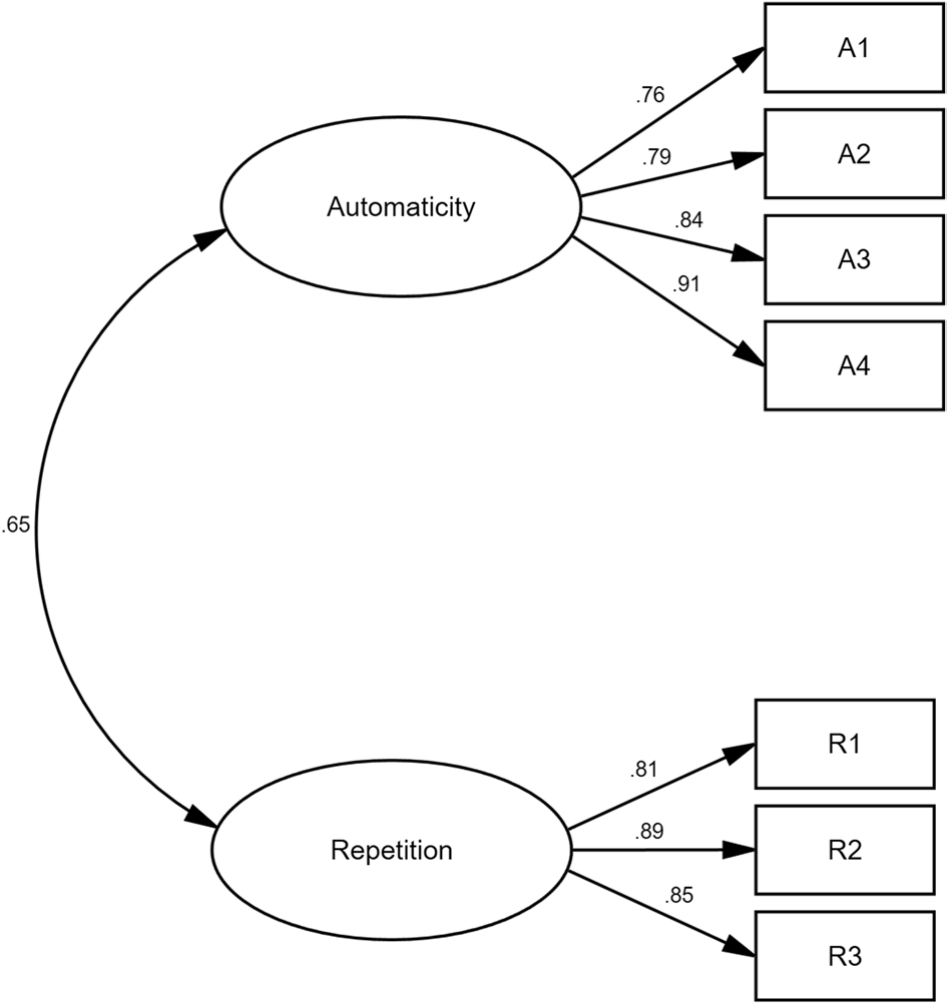
Confirmatory Factor Analyses (CFA) Model.

The Cronbach’s α coefficients of automaticity, repetition and the whole scale were all greater than 0.8, which indicated that the scale had great reliability. The factor load of each item to each dimension which was greater than 0.6 (*p* < 0.001), Average Variance Extracted value (AVE) and Construct Reliability (CR) of two dimensions which were greater than 0.5 and 0.8 respectively demonstrated that each item could be well aggregated in each dimension and the scale had great aggregation validity. The square roots of AVE in both dimensions were greater than the correlation coefficient between repetition and automaticity, which means the scale had great discriminative validity (Table 2). The Confirmatory Factor Analyses (CFA) model fitted the data and has been shown in Fig. 2 (χ^2^ = 21.250, DF = 13, RMSEA = 0.072, CFI = 0.985, GFI = 0.956).

**Table 2:** Results of Exploratory Factor Analysis (EFA) and Cronbach’s αcoefficient.

	KMO & Bartlett	Communalities	Items	AVE	CR	Correlation	Total	Cumulative %	Cronbach’s α coefficient
SREHI	KMO = 0.949***	Automaticity	4	0.684	0.896	0.645***	8.352	78.77%	0.894
		Repetition	3	0.724	0.887		1.1		0.915
		The scale	7						0.914

***Significant at the 0.001 level.

### Statistical analyses

Data was analyzed by SPSS 22.0 (IBM Inc., Chicago, IL, USA) and AMOS 23.0 (IBM Inc., Chicago, IL, USA). Specifically, Principal Component Analysis (PCA) was used for Exploratory Factor Analysis (EFA). Cronbach’s alpha (α) coefficients and Confirmatory Factor Analysis (CFA) were conducted to evaluate the reliability and construct validity of SREHI. The last but the most important was the test of mediation carried out by Structural Equation Modeling (SEM). To make sure the precondition of the mediating role, descriptive statistics and Pearson correlation coefficients were acquired in advance. After that, SEM was constructed to evaluate the mediating effects of demand and/or satisfaction by Asymptotically Distribution-Free (ADF) and BOOTSTRAP. The inferential statistics (i.e. significance, standardized regression coefficient and confidence interval) was to estimate path coefficient and mediation effect^42^.

Notably, due to sensitivity of chi-square χ^2^ to sample size^43,44^ and a variety of influencing factors to model fit^45^, multiple indexes would be reported. Of concern was the fact chi-square χ^2^ was heavily impacted by sample size and the model fit indexes were subsequently impacted by chi-square χ^2^. To attenuate the impact, Bollen-Stine bootstrap 5000 times was employed, chi-square χ^2^ (Bollen-Stine) and *p* (Bollen-Stine bootstrap) were reported^46^. Likewise, ADF would be carried out with a large sample size and non-normal distribution^47^. The level of significance statistically was set at *p* < 0.05 with two-tailed.

### Ethical approval information

The study had been approved by the Institional Review Board for Human Research Protections at Shanghai Jiao Tong University, China (H20200431I) and accorded with the guidelines of the Declaration of Helsinki.

## Result

### Descriptive statistics

Mean (M), Standard Deviation (SD), range (MIN & MAX) and Cronbach’s alpha of each item and dimension were shown in Table 3.

**Table 3:** Descriptive and Cronbach’s αcoefficient of Each Item and Dimension.

	M	SD	MIN	MAX	Cronbach’s α
Physical Exercise Behavior	4.34	0.88	1	5	
Demand	4.03	1.02	1	5	
Satisfaction	3.79	1.02	1	5	
Physical Exercise Habit	22.82	6.54	7	35	0.921
Repetition	10.91	2.94	3	15	0.914
R 1	3.71	1.04	1	5	
R 2	3.51	1.07	1	5	
R 3	3.69	1.07	1	5	
Automaticity	11.91	4.11	4	20	0.889
A 1	2.99	1.22	1	5	
A 2	2.66	1.17	1	5	
A 3	3.21	1.18	1	5	
A 4	3.06	1.17	1	5	

College students generally had physical exercise behavior (4.34 ± 0.88), whereas the stability of their physical exercise habits (22.82 ± 6.54) was only at a moderate intensity. Further, their physical exercise habits were relatively attributed more to repetition (10.91 ± 2.94) than automatic response (11.91 ± 4.11). There was a strong willingness for individuals to do physical exercise again for the same demand (4.03 ± 1.02). In light of satisfaction of their physical exercise experiences (3.79 ± 1.02), it was only at a moderate level statistically.

### Correlation analysis

The correlations among variables were presented in Table 4. Physical exercise behavior was positively correlated with physical exercise habits, demand and satisfaction (*r* = 0.373, *p* < 0.01; *r* = 0.478, *p* < 0.01; *r* = 0.464, *p* < 0.01), respectively. Demand was also positively associated with satisfaction and physical exercise habit (*r* = 0.501, *p* < 0.01; *r* = 0.368, *p* < 0.01), respectively. Satisfaction was positively associated with physical exercise habit (*r* = 0.562, *p* < 0.01). All correlations were significant and met the premise of the mediation hypothesis^48^.

**Table 4:** The correlations among variables.

	1	2	3	4
1. Physical Exercise Behavior	1			
2. Demand	0.478**	1		
3. Satisfaction	0.464**	0.501**	1	
4. Physical Exercise Habit	0.373**	0.368**	0.562**	1

**Significant at the 0.01 level.

### Testing of mediation models

**Figure 3:**
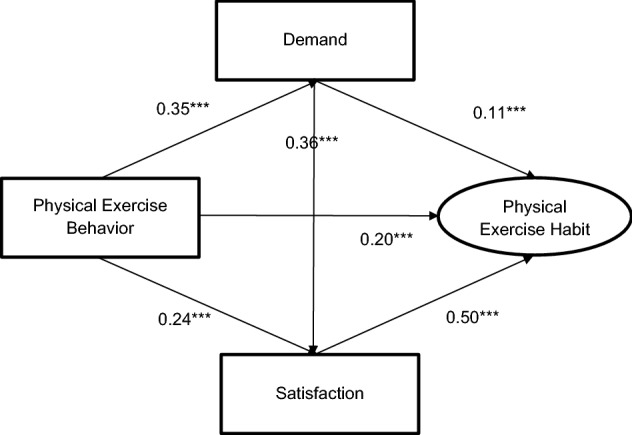
Path of Mediation mode. Note. *** is significant at the 0.001 level.

By meeting predetermination, SEM was constructed. Demand and satisfaction were added into physical exercise behavior—physical exercise habit path as mediators, and standardized regression coefficients of each path were obtained (χ^2^ = 932.04, χ^2^ (Bollen-Stine) = 33.115, *p* (Bollen-Stine bootstrap) < 0.001, DF = 32, RMSEA = 0.094, CFI = 0.701, GFI = 0.883) (Fig. 3). According to Fig. 3, all standardized regression coefficients reached significant levels (*p* < 0.05). Physical exercise habits could be positively predicted by physical exercise behavior, satisfaction and demand (*β* = 0.20, *p* < 0.001; *β* = 0.50, *p* < 0.001; *β* = 0.11, *p* < 0.001), respectively. Satisfaction could be positively predicted by physical exercise behavior and demand (*β* = 0.24, *p* < 0.001; *β* = 0.36, *p* < 0.001), respectively. Demand could be positively predicted by physical exercise behavior (*β* = 0.35, *p* < 0.001).

At the same time, BOOTSTRAP with 5000 times running and 95% CI were calculated. Since BOOTSTRAP CIs of direct, indirect and total effects did not contain 0 and were deemed as statistically significant, the partial mediation effect was consequently considered as existence. Based on Fig. 3 and Table 5, direct effect was 0.196; indirect effect was 0.221; and total effect was 0.418.

**Table 5:** Results of path analysis.

Path	Estimate	Bootstrap SE	Bias-corrected			Percentile			Effect
			Lower	Upper	*p*	Lower	Upper	*p*	
PEB → D → PEH	0.038	0.009	0.022	0.058	***	0.021	0.057	***	9.09%
PEB → S → PEH	0.121	0.013	0.097	0.148	***	0.096	0.147	***	28.95%
PEB → D → S → PEH	0.062	0.008	0.047	0.078	***	0.048	0.078	***	14.83%
INDIRECT EFFECT	0.221	0.018	0.186	0.258	***	0.185	0.258	***	52.87%
DIRECT EFFECT	0.196	0.029	0.139	0.253	***	0.139	0.255	***	46.89%
TOTOL EFFECT	0.418	0.031	0.351	0.475	**	0.354	0.477	***	1

Further decomposing the indirect effects, the indirect effect of demand (i.e. physical exercise behavior—demand—physical exercise habit) was 0.038, accounting for 9.09% mediation; the indirect effect of satisfaction (i.e. physical exercise behavior—satisfaction—physical exercise habit) was 0.121, accounting for 28.95% mediation; and the indirect effect of demand and satisfaction (i.e. physical exercise behavior—demand—satisfaction—physical exercise habit) was 0.062, accounting for 14.83% mediation. Hence, physical exercise behavior could develop into a physical exercise habit through single mediators of demand or satisfaction, or a chain mediator from demand to satisfaction. Hypotheses 1, 2 and 3 were confirmed.

PEB = physical exercise behavior; D = demand; S = satisfaction; PEH = physical exercise habit.

**Significant at the 0.01 level; *** is significant at the 0.001 level.

## Discussion

The aim of this research was to examine the formation processes of physical exercise habits. Results showed that university or college students generally had physical exercise behaviors, but the stability of their physical exercise habits was only at moderate intensity, that is, individuals’ physical exercise behaviors did not develop into physical exercise habits completely. Therefore, how to develop physical exercise behaviors into physical exercise habits was truly the matter. Given the limitations of previous theories in these topics and the nature of physical exercise behaviors (habits), the Model of Physical Exercise and Habit Formation was carried out. Results showed that physical exercise behaviors could develop into physical exercise habits through a direct path, single mediators of demand or satisfaction, or a chain mediator from demand to satisfaction.

What should be considered first in this study was the measurement of physical exercise habits. The counting of past frequencies, like "In the past… How many times have you done… ", was the most common and relatively reasonable measurement of a habit in literatures^19^, but a researcher pointed out that frequency could not reflect habits accurately^49^. Schwarz put forward that respondents might find it hard to recall what happened in a long time (e.g. how many times have you done… in the past… months or years), and they might not be absolutely clear about the terms (e.g. exercise intensity)^50^. To remedy the issues underlying the measurements, SRHI by Verplanken and Orbell was employed^51^ and it has been re-evaluated into a variety of fields^52–54^. What counts was the two dimensions (i.e. automaticity and repetition) extracted by SREHI (modified from SRHI) perfectly fitted the characteristics of physical exercise habits by Aarts et al. (i.e. automated response to a specific situation and tendency to repeat the same behavior)^10^. That is, SREHI might have more potential to satisfy what Arts believed and the Model elaborated.

Habit formation was deeply immersed in Behaviorism and in line with Learning theories^10^. According to the Stimulus—Respond (S-R) process, Thorndike proposed the experiment of hunger cats and the Law of Learning (i.e. Law of Readiness, Law of Exercise and Law of Effect) ^55^. What Thorndike stressed were: (1) the ready condition, that is the hunger condition of the cats or the need of doing exercise; (2) the repetition, even though it was revised later by himself that repetition was not adequate to intensify the relation between S and R; and (3) the satisfaction, which impact whether the behavior would maintain or not^56^. It seemed that the standpoint from Behaviorists accorded with Aarts’s and the Model. Indeed, the cognition process was still ignored, even if Thorndike prompted satisfaction. Evidence showed that engaging in a habitual behavior was different from simply engaging in a behavior frequently since the true habitual behavior was automatically activated^19^ and the influence of habit formation may be better attributed to automaticity^57^. Automaticity is, in essence, an unconscious state in which cognitive load is minimized^58^. The unconscious state was transformed from conscious thinking and calculating gradually, and they were continuous and unified rather than absolutely dichotomized^58^. A consensus that habits are formed gradually by reinforcing the connection between the context (or cues) and the existing behaviors ^59^. Further, the repetition of behaviors in a certain context could gradually increase the automaticity of the behaviors in that context^60^. Therefore, it can be said that repetition is to some extent a prerequisite for automation^61^.

Congruent with other scholars^19–21^, Aarts et al. believed that habit formation required repetition over a period of time as well^62^. The possibility of the same behavior occurring next time constituted an important bridge of repeating behaviors^10^ and the possibility could be triggered and activated by cues^63^. More practically, reappearance of the same needs, so-called cues, triggered the recurrence of physical exercise behaviors. It could therefore conclude that the reappearance of the same need was considered as the mediation of the cyclic execution of physical exercise behaviors. When the same need keeps appearing, cognition will be activated by repeated stimulations and the deliberation time will be shortened gradually, so that the conscious repeated behaviors could be transferred to the unconscious automatic responses^10^ and physical exercise habits may ultimately be formed. Similar findings went to the research by Kang et al. about the significance of needs in predicting exercise adherence^64^.

Emotional judgment of exercise experiences was a major and the strongest predictor of habit formation^65,66^, and the formation of physical exercise habits depended on the self-perception of past physical exercise experience. Positive attitudes required little or no reflective process^67^, and these positive attitudes could become unconsciously driven for the next behaviors in turn ^63^. Empirical studies showed that physical exercise habits could be formed through repeated satisfactory physical exercise experiences as well^41,68^. Starting with an enjoyable exercise experience could help with habit formation^20^; on the contrary, individuals would easily give up adherence to physical exercising if unexpected and negative difficulties and consequences resulted from previous exercise experiences^10^. For example, with the same 30-min physical exercise experience, some people may feel delighted and cheerful with the decision to do physical exercise again, other people may feel torturous and painful with the decision to give up experiencing one more time to avoid misery. To this end, it appears that satisfaction could be deemed as a bridge between past, present and future exercise experiences until physical exercise habits form.

Despite serving as a single mediator in the relationship between physical exercise behavior and physical exercise habit, need and satisfaction were not isolated. Neither of them could guarantee that participants would engage in physical exercise again for sure, but both of them do elevate the likelihood that participants prefer to do physical exercise next time. Therefore, demand and satisfaction could be a chain mediator affecting the relationship between physical exercise behavior and physical exercise habit. It was necessary to note that the path of the chain was from need to satisfaction rather than from satisfaction to need due to the Information Processing Model by Gagne^69^, even though both paths were significant statistically. To be specific, the perception of physical exercise experience (i.e. satisfaction) was encoded into long-term memory and working memory of the perception (i.e. satisfaction) was drawn out only after encountering cues (i.e. demands) and then physical exercise behavior would be driven again^69^.

Overall, physical exercise behaviors could develop into physical exercise habits through a direct path, single mediators of demand or satisfaction, or a chain mediator from demand to satisfaction. Based on the Model of Physical Exercise and Habit Formation, it appears that when physical exercise behavior exists, the appearance of demand is treated as a clue to stimulate memory and that the satisfaction may be feedback to prompt the next exercise-choosing process. Then, after repeating this process, responses to these clues will occur automatically with cognitive shortcuts and physical exercise behaviors will form eventually as physical exercise habits.

Implications should be supplied to the public and physical exercise educators or instructors. With regard to enhancing satisfaction, utilization of high-tech could make it more readily accessible. Research showed that Virtual Reality (VR) exercise made physical exercise interesting and could relieve negative moods, such as fatigue and tension^70^. Furthermore, the promotion of moods could be conducive to endurance^71^ and adherence^72^ in physical exercising. Unlike satisfaction, demand as a situational cue is not predictable and controllable. But if Physical Literacy (PL) could be promoted, the individuals would have the knowledge on how to fulfill their demands with physical exercise (e.g. how to lose weight with physical exercise) and the capability to participate in physical exercise (e.g. how to swim)^73^, which are essential to initial a physical exercise behavior. As discussed, it might be helpful to cultivate physical exercise habits by improving PL to find out individuals’ demands to participate in physical exercise and using high-tech to enhance satisfaction, thereby increasing the possibility of behaviors repetition and automaticity.

Large and comprehensive samples as well as the perfectly Model-fitted measurement were the most eye-catching strengths of this study. Yet, limitations were still not to be ignored: (1) even though samples were from 34 provinces and religions in China, there was still a bias representation; (2) uncontrolling confounding factors, like grade or frequency of PE courses, might limited the validity to some extent; (3) self-reported questionnaire might be subjective in this study.

In the future, we strongly encourage that longitudinal study design could be conducted and mechanisms of physical exercise habits formation in neurocognition could be explored. If possible, socioeconomic information of extensive participants, such as jobs and social status, should also be taken into account in that the development and maintenance of positive physical exercise habits is not laboratory-oriented but needs to be extrapolated into reality. Interestingly, the reason why satisfaction plays an important role in physical exercise habits formation might be physiology-oriented, like the secretion of dopamine. Another possible phenomenon—exercise addiction—may emerge, when exercisers pursue satisfaction excessively. In that case, the boundary between physical exercise habit and exercise addiction and how to correct exercise addiction will also become mainstream in the future.

## Conclusion

Findings showed that physical exercise behaviors could develop into exercise habits through a direct path, single mediators of demand or satisfaction, or a chain mediator from demand to satisfaction. For the public and physical exercise educators or instructors, it might be helpful to cultivate physical exercise habits by improving PL to find out individuals’ demands to participate in physical exercise, and using high-tech to enhance satisfaction, thereby increasing the possibility of behaviors repetition and automaticity. Nevertheless, given bias representation, uncontrolling confounding factors and subjective measurements, rigorous and longitudinal study design should be considered. What is more, exercise addiction, a related possible phenomenon, should be noted in the future.

## Data Availability

The data presented in this article are available. The content related to the privacy of participants is not publicly available.
