# Publisher Correction: The mediating roles of demand and satisfaction in formation process of physical exercise habits among college students

**DOI:** 10.1038/s41598-022-07060-3

**Published:** 2022-02-14

**Authors:** Kun Wang, Jiali Qian, Jiayi Yang, Tianyi Ge, Zhizhong Li

**Affiliations:** Department of Physical Education, Shanghai Jiao Tong University, 204 Guangming Hall, NO. 800 Dongchuan Street, Minhang District, Shanghai, 200240 China

Correction to: *Scientific Reports* https://doi.org/10.1038/s41598-022-05602-3, published online 28 January 2022

The original version of this Article contained an error in the Affiliation, which was incorrectly given as ‘Department of Physical Education, Shanghai Jiao Tong University, 204 Guangming Hall, NO. 800 Dongchuan Street, Minhang District, Shanghai, 200240, USA’. The correct affiliation is listed below:

Department of Physical Education, Shanghai Jiao Tong University, 204 Guangming Hall, NO. 800 Dongchuan Street, Minhang District, Shanghai, 200240, China.

The original Article has been corrected.

